# Development of active packaging for the preservation of lyophilized pulp fruit

**DOI:** 10.1186/1753-6561-8-S4-P206

**Published:** 2014-10-01

**Authors:** Rejane Andrade Batista, Narendra Narain, Nilda de Fátima Ferreira Soares, Paula Judith Pérez Espitia, Eber Antonio Alves Medeiros, Patrícia Nogueira Matos

**Affiliations:** 1 Laboratory of Flavor & Chromatographic Analysis, PROCTA, Federal University of Sergipe, São Cristóvão, SE, Brazil; 2 Food Packaging Laboratory, Food Technology Department, Federal University of Viçosa, Viçosa, MG, Brazil

## Introduction

Innovative technologies are needed to maintain food freshness, quality and safety, while reducing concentrations of chemical additives. This work aimed the development of an active packaging material to preserve lyophilized tropical fruits pulp powder and to study the stability provided by the active packaging.

## Methodology

Active packaging material was low density polyethylene (LDPE) film incorporated with Tinuvin 326(UV absorber) and iron powder in different concentrations (0-0.5% and 0-15% w/w, respectively). Eleven treatments were performed according to the central composite design (CCD) in order to evaluate changes in mechanical, physical and barrier properties of films. Moreover, the influence of selected optimal active packaging on the stability of lyophilized tropical fruits pulp was assessed.

## Results

The incorporation of Tinuvin 326 in LDPE did not alter the physical and mechanical properties of the film. Tinuvin 326 promoted barrier activity to UV light with increasing concentrations while maintaining film transparency. Thus, incorporation of 0.5% Tinuvin 326 and 7.5% iron was considered the most suitable conditions according to the Response Surface Methodology, for the physicochemical and microbiological characteristics of lyophilized pulp. Moreover, the lyophilized tropical fruits pulp powder was stable for most of the analyzed parameters during storage of the products.

## Conclusions

The developed packaging maintained the chemical integrity, physical properties and resistance to microbial growth of the packaged food, during the storage period. Due to their nature, this package may be produced on industrial scale for lyophilized products preservation, replacing laminated packaging. Therefore, developed active packaging has potential for the preservation of lyophilized fruits pulp powder.

